# Autoantibodies combined with systemic inflammation markers for predicting bone metastases in non-small cell lung cancer patients

**DOI:** 10.3389/fimmu.2026.1747604

**Published:** 2026-05-26

**Authors:** Song Cheng, Dabin Chen, Rongrong Du, Chao Wang, Jiawen Xian, Liyuan Liu, Hong Wan, Ting Ye

**Affiliations:** 1 Department of Laboratory Medicine, the Affiliated Hospital of Southwest Medical University, Sichuan, China; 2 Department of Thoracic Surgery, the Affiliated Hospital of Southwest Medical University, Sichuan, China

**Keywords:** anti-ENAs, autoantibodies, bone metastases, nomogram, non-small cell lung cancer (NSCLC), systemic inflammation markers

## Abstract

**Objective:**

This study aims to develop and validate a nomogram model that integrates autoantibodies and systemic inflammation markers to predict the risk of bone metastases in patients with non-small cell lung cancer (NSCLC). Additionally, we propose a novel approach for risk stratification and adjunctive assessment of bone metastases in NSCLC patients, aiming to support clinical decision-making.

**Methods:**

This retrospective study analyzed 323 NSCLC patients treated at the Affiliated Hospital of Southwest Medical University from January 2020 to July 2024. Comprehensive clinical, laboratory, and imaging data were collected. Key predictors included histology, TNM stage, ANA fluorescence patterns, anti-extractable nuclear antigens (anti-ENAs), SIRI, LWR, and anti-AMA-M2. Least absolute shrinkage and selection operator (LASSO) regression was used for feature selection, and variables with non-zero coefficients were incorporated into a nomogram. The model was validated internally using receiver operator characteristic curve (ROC) analysis, calibration curves, and decision curve analysis (DCA). The incremental value of novel biomarkers was assessed using NRI and IDI.

**Results:**

Seven variables were retained in the final nomogram, including histology, TNM stage, anti-ENAs, SIRI, LWR, anti-AMA-M2, and ANA fluorescence pattern. The nomogram demonstrated good discriminatory ability, with the receiver operating characteristic curve (AUC) of 0.921 (95% CI: 0.887-0.955) in the training cohort and 0.870 (95% CI: 0.795-0.945) in the validation cohort. Calibration plots showed good agreement between predicted and observed outcomes. Decision Curve Analysis (DCA) indicated that the nomogram provided a higher net benefit compared to “treat-all” and “treat-none” strategies across a range of threshold probabilities. The inclusion of novel biomarkers significantly improved the model’s predictive performance, as evidenced by continuous NRI (0.822, *P<* 0.001) and IDI (0.121, *P*<0.001).

**Conclusion:**

The nomogram developed in this study offers a reliable tool for individualized risk prediction of bone metastasis in NSCLC patients. Incorporating autoantibody and inflammation-related biomarkers significantly enhances the predictive performance, which may help in risk stratification and early intervention.

## 1 Introduction

Despite therapeutic advancements including targeted therapies and immunotherapies that have markedly enhanced survival outcomes, lung cancer persists as the leading cause of cancer-related mortality globally, constituting a major public health challenge (1–3). Lung cancer is primarily classified into two major types based on the histopathological characteristics of tumor cells: small cell lung cancer (SCLC) and non-small cell lung cancer (NSCLC). NSCLC accounts for approximately 85% of all lung cancer cases worldwide (4), and 20% to 40% of NSCLC patients develop bone metastases to varying degrees during cancer progression, with a median survival of only 6–10 months (5–7). Bone metastases can lead to skeletal-related events (SREs), such as bone pain, pathological fractures, spinal cord compression, and hypercalcemia (7), which significantly reduce patient mobility and quality of life, as well as impose substantial psychological and financial burdens. Timely identification of patients at high risk for bone metastases may facilitate earlier clinical intervention, potentially reducing disease burden and improving patient outcomes.

Current diagnostic approaches for NSCLC bone metastases primarily rely on symptomatic presentation and radiographic imaging. However, imaging-confirmed metastases are typically associated with advanced disease stages and poor prognoses (8–10). Recent studies have identified several biological indicators, including bone resorption markers, bone formation markers, and bone metastasis-related signaling markers, as potential predictors of bone metastasis in lung cancer (11, 12). Chai et al. (11) conducted a systematic review of these markers, highlighting bone resorption-related markers (e.g., N-terminal telopeptide [NTx]/C-terminal telopeptide [CTx] and C-terminal telopeptide of type I collagen [CTx-I]), bone formation-related markers (e.g., total serum alkaline phosphatase [ALP]/bone-specific alkaline phosphatase [BAP], osteobridging protein [OP], and osteocalcin [OS]), and bone metastasis-related signaling markers such as EGFR/KRAS/ALK for the prediction and prognosis of lung cancer bone metastasis and its therapeutic value. Nevertheless, methodological inconsistencies and heterogeneous findings have hindered the establishment of standardized diagnostic criteria. Additionally, the high cost of these assays limits their widespread use in clinical practice, particularly in resource-limited settings. This highlights the urgent need for developing validated predictive models with optimized cost-effectiveness.

Autoantibodies are usually antibodies produced against components of one’s own cells, tissues, and organs, serving as critical biomarkers for autoimmune diseases (13). In healthy individuals, these antibodies are typically absent or present at very low concentrations in the blood (14). However, in cancer patients, the positive detection rate of certain autoantibodies is significantly elevated (15, 16). Emerging evidence suggests that autoantibodies may play a role in the development of malignant tumors (15, 17, 18). For instance, Böckle et al. (19) demonstrated that Ro/SS-A antibody positivity can precede the clinical diagnosis of malignancy by a considerable period. Similarly, Gauderon et al. (17) reported that antinuclear antibodies with a nucleolar phenotype are associated with cancer presence. These studies suggest that specific autoantibody titers and fluorescence patterns may contribute to tumor detection. Furthermore, autoantibodies have been associated with tumor prognosis (20, 21) and are widely investigated as potential biomarkers for cancer detection, risk assessment (22–24), and prognostic monitoring (24–26). Additionally, autoantibodies may be implicated in cancer metastasis. For example, Adina Thoelke et al. (27) observed that a patient developed high seropositivity for Jo-1 antibodies two months after being diagnosed with metastatic melanoma, suggesting that elevated autoantibody titers may signal cancer metastasis. These findings imply that autoantibodies in cancer patients could serve as potential markers for distinguishing metastatic from non-metastatic cancers. Notably, no studies have yet explored the relationship between autoantibodies and bone metastasis in non-small cell lung cancer, highlighting a promising area for future research.

Inflammatory response plays a critical role in the tumor microenvironment and is closely associated with tumorigenesis, progression, invasion, and metastasis (28, 29). Tumor cell proliferation can stimulate the growth of inflammatory cells, which subsequently alter the tumor microenvironment and promote angiogenesis through the release of inflammatory factors, thereby facilitating tumor metastasis and immune evasion (29). Bone marrow, as a major reservoir of dendritic cells, macrophages, myeloid cells, and various T-cell subpopulations, functions as an immune regulatory organ that modulates the immune system and facilitates the transport of immune cells (30). Studies have demonstrated that mononuclear macrophages promote the growth of bone metastases in breast cancer patients (31), while patients with metastatic lung cancer exhibit significantly lower absolute lymphocyte counts (ALC) and higher absolute neutrophil counts (ANC) (32). Consequently, investigating changes in inflammatory markers during cancer progression offers novel insights for identifying diagnostic and prognostic tumor markers. Systemic inflammation markers such as the lymphocyte-monocyte ratio (LMR), platelet-lymphocyte ratio (PLR), neutrophil-lymphocyte ratio (NLR), systemic immune-inflammation index (SII), and systemic inflammatory response index (SIRI) have garnered significant attention in recent years. Due to their cost-effectiveness and ease of detection, these markers have been frequently reported to correlate with the development of non-small cell lung cancer (NSCLC) (33–36). Alterations in these indicators may not only reflect the systemic immune and inflammatory status of NSCLC patients but could also be linked to the development and progression of bone metastases.

In this study, we collected easily accessible clinical data, including autoantibody results and routine blood markers, to identify risk factors associated with bone metastases in NSCLC patients. Our objective is to identify high-risk groups for bone metastases among patients with NSCLC, thereby supporting timely clinical intervention and potentially improving patient quality of life and long-term outcomes.

## 2 Materials and methods

### 2.1 Analysis of NSCLC data in GEO public database

The NSCLC dataset, including mRNA expression and clinical information, was obtained from GEO Database. The original data from GEO was normalized and analyzed by the edgeR analysis method. After normalizing the GEO dataset, we distinguished between bone metastases group and non-metastases group according to the mRNA expression level. The differences of GO functional and KEGG pathway enrichment between the two groups were obtained.

### 2.2 Study population

This retrospective study analyzed 323 NSCLC cases treated at the Affiliated Hospital of Southwest Medical University between January 2020 and July 2024. Comprehensive clinical and laboratory data were systematically collected through the hospital’s electronic medical record system. The study was approved by the Institutional Review Board of the Affiliated Hospital of Southwest Medical University (Approval No. KY2024460), and the requirement for informed consent was waived due to the retrospective nature of the study.

Inclusion criteria: (1) histopathologically or cytologically confirmed NSCLC diagnosis, including adenocarcinoma and squamous cell carcinoma subtypes; (2) absence of concurrent malignancies; (3) confirmed bone metastasis by either histopathological examination or characteristic imaging findings, with magnetic resonance imaging (MRI) serving as the primary imaging modality for evaluation (37). All imaging results were independently reviewed by at least two experienced radiologists. (4) No prior treatment at enrollment, defined as no history of anti-tumor therapy, including surgery, radiotherapy, chemotherapy, targeted therapy, or immunotherapy.

Exclusion criteria: (1) non-NSCLC lung cancer histology; (2) incomplete clinical data; (3) concurrent bone metabolic disorders (e.g., hyperparathyroidism, severe osteoporosis, or chondromalacia); (4) pregnancy or lactation status; (5) hematological disorders; and (6) autoimmune diseases or active severe infections.

Based on epidemiological data indicating a 30%–40% incidence of bone metastasis in patients with NSCLC (4), the sample size was calculated using the standard formula (36): $\text{n}=\frac{Z_{1-\frac{\alpha }{2}}^{2}\times P\times \left(1-P\right)}{E^{2}}$, where $Z_{1-\frac{\alpha }{2}}$ was set at 1.96, *P* was assumed to be 0.30, and *E* was set at 0.05.

In this study, the proportion of missing data for all variables was less than 5%, which was considered low. Missing values in continuous variables were imputed using the median, whereas missing values in categorical variables were imputed using the mode.

### 2.3 Detection of antinuclear antibodies and anti-extractable nuclear antigens

ANA and anti-ENAs were detected using the German EUROIMMUN anti-nuclear antibody kit and the extractable nuclear antigen antibody (IgG) detection kit, respectively. Negative and positive controls were set for each experiment to ensure the accuracy of the results.

#### 2.3.1 ANA detection

Venous blood samples (3–5 mL) were collected from fasting subjects and processed by centrifugation at 3000 rpm for 10 minutes to obtain serum. ANA detection was performed using indirect immunofluorescence (IIF) technique with HEP-2 cells and primate liver tissue sections as dual substrates, following the manufacturer’s protocol. Fluorescence patterns were systematically analyzed and categorized according to established criteria, including homogeneous, speckled, nucleolar, centromere, nuclear membrane, cytoplasmic, and Golgi patterns. The initial screening dilution was set at 1:100, with negative results defined by the absence of characteristic nuclear fluorescence patterns. Positive samples were further serially diluted (1:100, 1:320, 1:1000, and 1:3200) to determine antibody titers.

#### 2.3.2 anti-ENAs detection

Venous blood samples (3–5 mL) were collected from fasting participants and centrifuged at 3000 rpm for 10 minutes to isolate the serum layer. Using the immunoblotting technique (IBT), immunoglobulin G (IgG) antibodies targeting 14 specific antigens were analyzed, including nRNP, Sm, SS-A (including Ro-52), SS-B, Scl-70, Jo-1, CENP B, PCNA, ds-DNA, nucleosomes, histones, ribosomal P protein (RIB-P), and anti-AMA-M2. All procedures strictly followed the manufacturer’s protocols. A positive result for any antibody in this panel was classified as a positive anti-ENA test outcome. These 14 specific antigens were treated as binary variables (negative, positive) in the model analysis.

### 2.4 Complete blood count

Participants fasted for 12 hours prior to venous blood collection (3–5 mL), which was performed in the morning using sterile vacuum tubes. Blood samples were immediately analyzed using a BC-6800 automated hematology analyzer (Mindray, Shenzhen, China) with manufacturer-matched reagents. Measured parameters included: white blood cell count (WBC, 10^9/L), platelet count (PLT, 10^9/L), neutrophil count (NEU, 10^9/L), lymphocyte count (LYM, 10^9/L), monocyte count (MONO, 10^9/L), basophil count (BASO, 10^9/L), and eosinophil count (EOS, 10^9/L).

All assays were performed by the same team of certified laboratory technicians. Standard operating protocols were strictly adhered to throughout the analytical process, with quality control according to CNAS-CL02: Accreditation Criteria for the Quality and Competence of Medical Laboratories (ISO15189:2012, Medical laboratories - Requirements for quality andcompetence, IDT).

### 2.5 Calculation of the systemic inflammation markers

Systemic inflammatory markers were calculated as follows:

LMR = Lymphocyte count (10^9/L)/Monocyte count (10^9/L).
NLR = Neutrophil count (10^9/L)/Lymphocyte count (10^9/L).
PLR = Platelet count (10^9/L)/Lymphocyte count (10^9/L).
LWR = Lymphocyte count (10^9/L)/White blood cell count (10^9/L).
ELR = Eosinophil count (10^9/L)/Lymphocyte count (10^9/L).
BLR = Basophil count (10^9/L)/Lymphocyte count (10^9/L).
SII = Neutrophil count (10^9/L)×Platelet count (10^9/L)/Lymphocyte count (10^9/L).
SIRI = Neutrophil count (10^9/L)×Monocyte count (10^9/L)/Lymphocyte count (10^9/L).

### 2.6 Data analysis

The dataset was randomly divided into a training set and an internal validation set at a ratio of 7:3 using computer-generated random numbers in SPSS (version 26.0; IBM Corp., Armonk, NY, USA), with assignment based on numerical ranking. This random allocation helped reduce selection bias and supported the reliability of subsequent model development and validation. Baseline demographic characteristics were compared between the training and validation cohorts. Continuous variables were expressed as mean ± standard deviation for normally distributed data and as median (interquartile range) for non-normally distributed data. Group comparisons for continuous variables were performed using Welch’s two-sample t test or the rank-sum test, as appropriate. The definitions and coding of variables are presented in **Supplementary Table 1**. Candidate variables were first entered into a LASSO regression model for feature selection. Variables with non-zero coefficients selected by LASSO were all retained and incorporated into the nomogram. Multivariable logistic regression was subsequently performed to estimate the effect size of each selected variable, and the corresponding regression coefficients, odds ratios, and 95% confidence intervals were reported. No further variable elimination was performed based on the multivariable logistic regression results. The performance of the model was assessed using the ROC curve and calibration curve, with the AUC ranging from 0.5 (no discriminant) to 1 (complete discriminant). Internal validation was performed using bootstrap resampling to calculate a corrected concordance index (c-index). A calibration curve was plotted to assess the agreement between predicted probabilities and observed outcomes. A decision curve analysis (DCA) was also performed to determine the net benefit threshold of prediction. Results with *P<* 0.05 were considered significant. All statistical analyses were performed using SPSS 26.0 and R software (version 4.3.2).

## 3 Results

### 3.1 Expression and functional enrichment analysis of autoimmune-related genes in metastatic NSCLC patients

**Figure 1:**
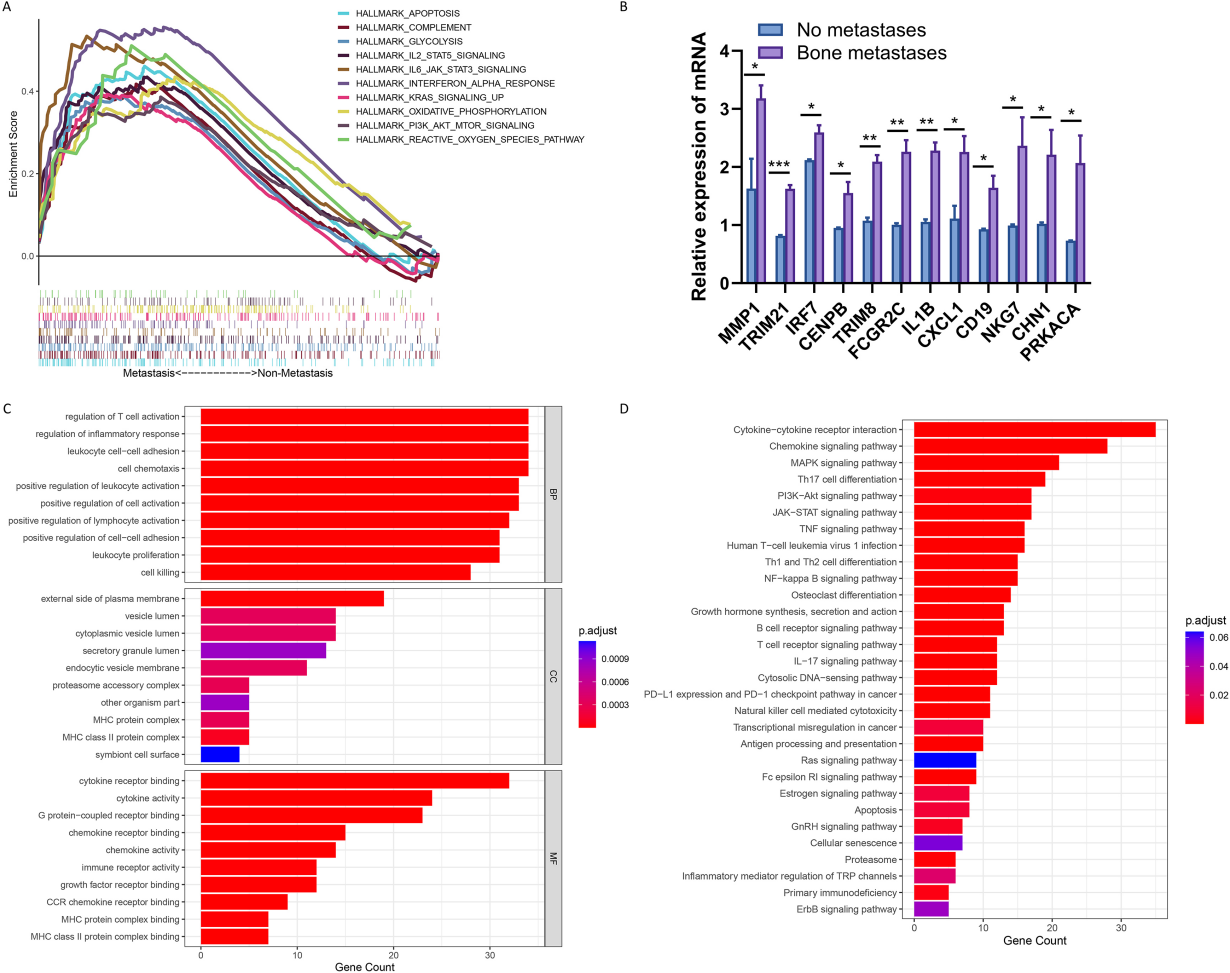
Expression levels of autoantibody-related and metastasis-related genes and functional enrichment analysis. **(A)** Gene set enrichment analysis (GSEA) showing Hallmark pathways enriched in the bone metastasis group compared with the non-metastasis group. **(B)** Relative mRNA expression levels of selected autoantibody-related and metastasis-associated genes in the non-metastasis and bone metastasis groups. C-D. Gene Ontology (GO). and Kyoto Encyclopedia of Genes and Genomes (KEGG) pathway enrichment analysis of differentially expressed autoantibody-related genes. **(C)** GO. **(D)** KEGG.

Using GEO datasets, we first compared the global transcriptomic profiles between bone metastatic and non-metastatic NSCLC patients. As shown in **Figure 1A**, GSEA revealed that several Hallmark pathways were significantly enriched in the bone metastasis group. Among them, apoptosis, glycolysis, oxidative phosphorylation, KRAS signaling, PI3K/AKT/mTOR signaling, and reactive oxygen species pathways were closely associated with tumor progression and metastatic activity, whereas complement activation, IL2/STAT5 signaling, IL6/JAK/STAT3 signaling, and interferon-α response were mainly related to immune activation and inflammatory regulation.

Based on the enrichment of and immune-related and metastasis-related pathways, we further focused on genes associated with autoantibody responses and metastasis. As shown in **Figure 1B**, metastasis-related genes, including MMP1, CXCL1, NKG7, CHN1, and PRKACA, were upregulated in the bone metastasis group. Notably, several autoantibody-related genes, including TRIM21, IRF7, CENPB, TRIM8, FCGR2C, IL1B, and CD19, were also significantly increased in patients with bone metastasis. These results suggest that bone metastasis in NSCLC may be accompanied not only by enhanced metastatic potential but also by activation of autoimmunity-related molecular signals.

To further characterize the biological functions of these differentially expressed autoantibody-related genes, we performed GO enrichment analysis using autoantibody-related genes screened from GeneCards that were differentially expressed between the bone metastasis and non-metastatic groups. These genes were mainly enriched in immune- and inflammation-related biological processes, including regulation of T cell activation, inflammatory response, leukocyte cell–cell adhesion, cell chemotaxis, and positive regulation of leukocyte activation. In addition, enrichment was observed in cytokine receptor binding, cytokine activity, G protein–coupled receptor binding, chemokine receptor binding, and immune receptor activity (**Figure 1C**).

Consistently, KEGG pathway analysis showed that these differentially expressed autoantibody-related genes were significantly enriched in multiple immune-inflammatory pathways, including cytokine–cytokine receptor interaction, chemokine signaling pathway, MAPK signaling pathway, PI3K–Akt signaling pathway, JAK–STAT signaling pathway, and TNF signaling pathway (**Figure 1D**). Together, these findings indicate that autoantibody-related genes are upregulated in bone metastatic NSCLC and may participate in immune activation and inflammatory signaling associated with metastatic progression.

**Figure 2:**
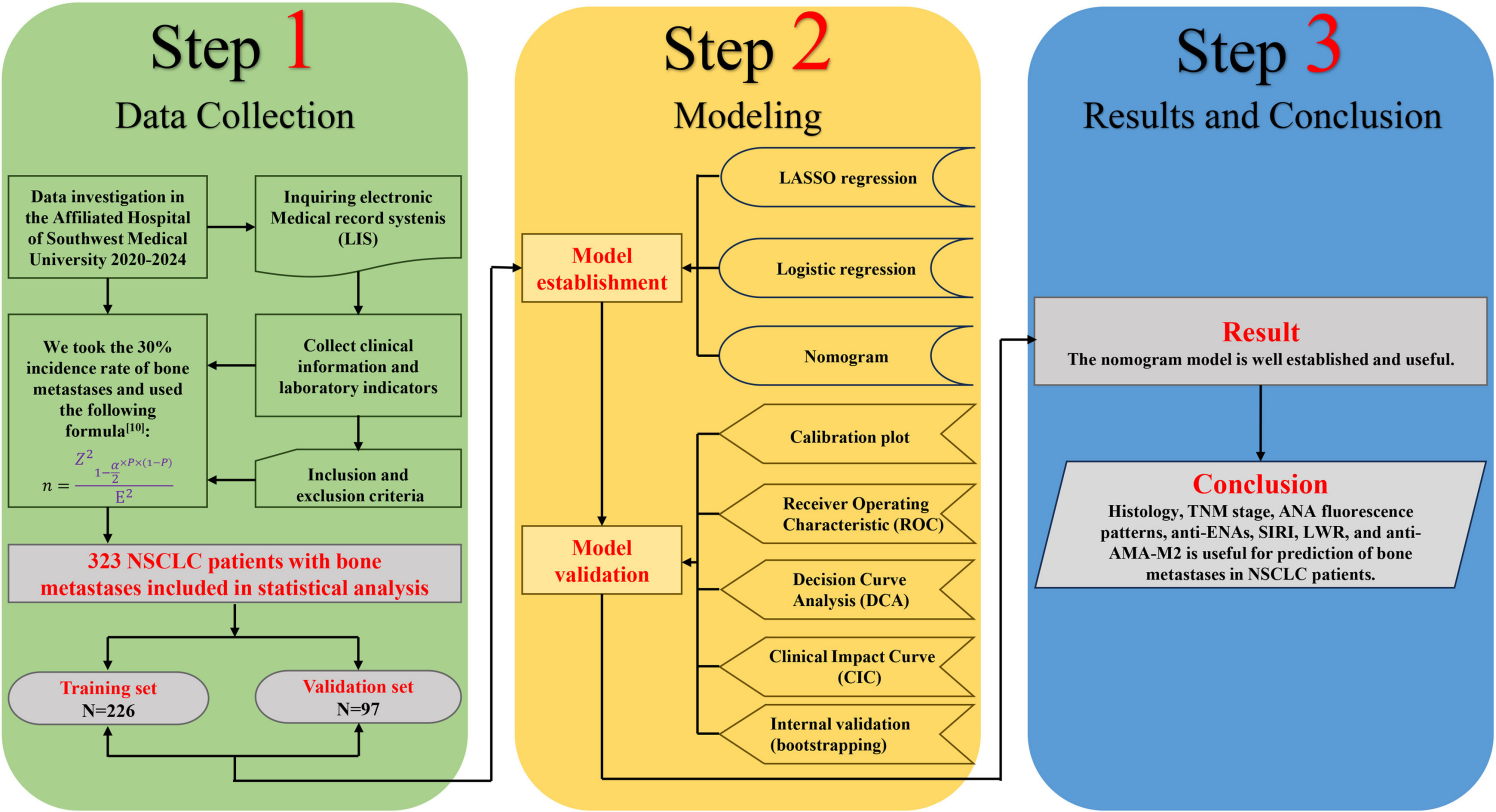
Flow chart of nomogram model construction.

Based on these findings, we developed a predictive model combining autoantibody-related variables, systemic inflammation markers, and clinicopathological characteristics. The study design and modeling workflow are shown in **Figure 2**.

### 3.2 Significant differences in ANA and anti-ENAs positivity were observed between the bone metastasis and non-bone metastasis groups

**Figure 3:**
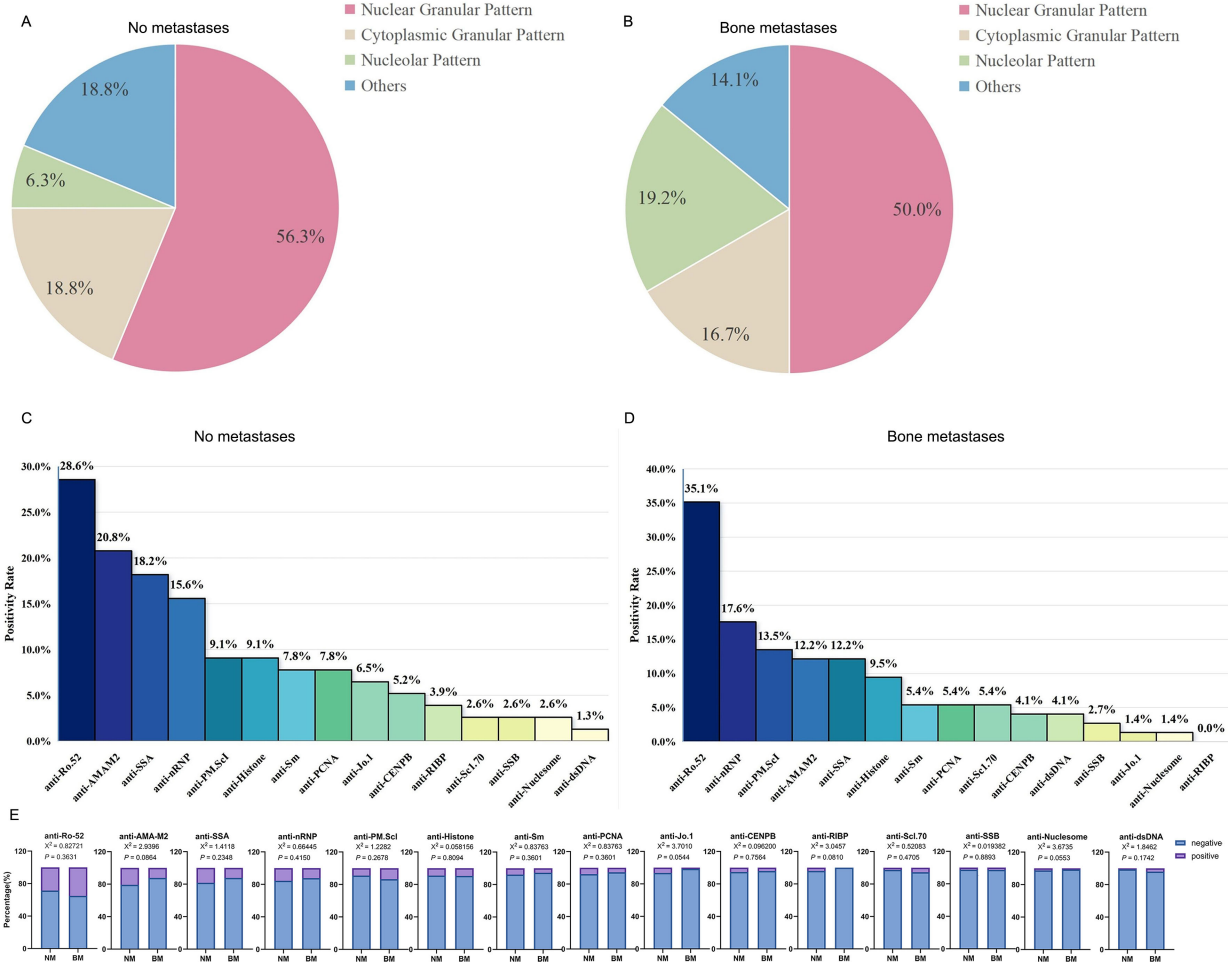
Autoantibody characteristics. **(A)** ANA fluorescence pattern distribution in bone metastasis patients. **(B)** ANA fluorescence pattern distribution in none bone metastasis patients. **(C)** Characteristics of anti-ENAs in bone metastasis patients. **(D)** Characteristics of anti-ENAs in none bone metastasis patients. **(E)** Comparison of anti-ENA antibody positivity between the non-metastatic (NM) and bone metastasis (BM) groups.

The distribution patterns of ANA and anti-ENAs are presented in **Figures 3A, B**. In the bone metastasis set (115/323, 35.6%), ANA positivity was detected in 48 patients (41.7%), demonstrating predominant nuclear granular patterns (27/48, 56.3%), followed by cytoplasmic granular patterns/others (9/48, 18.8%). Anti-ENA seropositivity occurred in 77 patients (66.9%), with the most prevalent specificity being anti-Ro-52 antibodies (22/77, 28.6%), succeeded by anti-AMA-M2 antibodies (16/77, 20.8%).

In **Figures 3C, D**, within the non-metastatic set (208/323, 64.4%), 78 patients (37.5%) exhibited ANA positivity, predominantly displaying nuclear granular patterns (39/78, 50.0%) and nucleolar patterns (15/78, 19.2%). Anti-ENA positivity was observed in 74 patients (35.5%), dominated by anti-Ro-52 antibodies (26/74, 35.1%), followed by anti-nRNP antibodies (13/74, 17.6%).

As shown in **Figure 3E**, comparisons of individual anti-ENA antibody positivity rates between the bone metastasis and non-metastatic groups revealed no statistically significant differences for all antibodies. Although no individual anti-ENA specificity reached statistical significance, this single-marker comparison may not fully capture the combined predictive value of autoantibody-related features. Therefore, these variables were further incorporated into subsequent multivariable modeling analyses.

### 3.3 Baseline comparability between cohorts and feature selection in the training cohort

**Figure 4:**
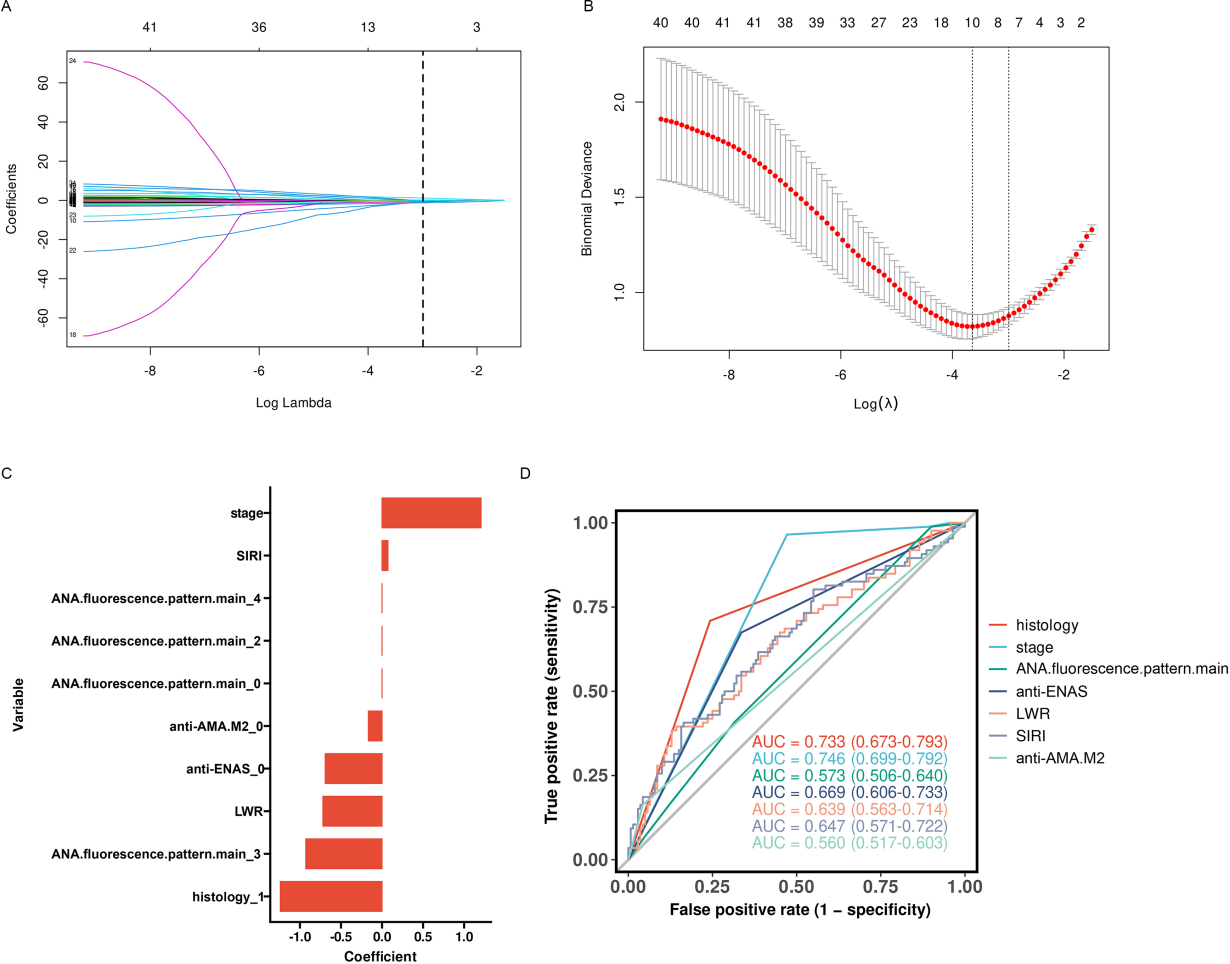
LASSO regression for feature selection and predictive performance of selected variables. **(A)** LASSO coefficient profiles of candidate variables. **(B)** Ten-fold cross-validation plot for selection of the optimal penalty parameter λ in the LASSO model. **(C)** Coefficients of variables retained at the optimal λ value. **(D)** Receiver operating characteristic (ROC) curves of the variables selected by LASSO regression.

Clinical characteristics and laboratory indexes were retrospectively analyzed for both sets. No significant differences in baseline demographics or laboratory indexes were identified between the training set (N=226) and internal validation set (N=97) (*P* > 0.05). These findings indicate adequate inter-cohort characteristic balance, thereby supporting the methodological robustness of subsequent predictive modeling in this NSCLC bone metastasis study. To identify potential predictors of bone metastasis, the following candidate variables were initially included in the original model: stage, histology, ANA fluorescence pattern, anti-ENAs, anti-AMA-M2, SIRI, RIB-P, gender, NLR, LWR, LYM, LMR, SII, PLR, NEU, nRNP, SS-A, SS-B, MONO, Sm, WBC, smoking history, Titer, EOS, BLR, PM.Scl, CENP B, Nucleosome, PLT, Ro-52, ELR, PCNA, BASO, ds-DNA, age, ANA, Sc1-70, Jo-1, and Histones. These variables were subsequently entered into a LASSO regression model in the training cohort. The coefficient profiles of the candidate variables are shown (**Figure 4A**), and the cross-validated binomial deviance plot for selection of the optimal penalty parameter λ is presented (**Figure 4B**). Based on the 1-SE criterion, the optimal λ value was selected, at which 7 predictors retained non-zero coefficients and were therefore included in the final model. The selected predictors and their corresponding coefficients are presented (**Figure 4C**), including histology, stage, anti-ENAs, SIRI, LWR, anti-AMA-M2, and ANA fluorescence pattern-related variables. Their predictive performance is further illustrated by ROC curve analysis, ROC analysis of these variables yielded AUC values greater than 0.5. (**Figure 4D**). Clinical stage showed the best predictive performance, with an AUC of 0.746 (95% CI: 0.699-0.792), followed closely by histology (AUC, 0.733; 95% CI: 0.673-0.793). Among the autoantibody-related markers, anti-ENAs achieved an AUC of 0.669 (95% CI: 0.606-0.733), whereas ANA fluorescence pattern and anti-AMA-M2 showed relatively limited discriminatory ability, with AUC values of 0.573 (95% CI: 0.506-0.640) and 0.560 (95% CI: 0.517-0.603), respectively. Among the inflammatory markers, SIRI and LWR yielded AUC values of 0.647 (95% CI: 0.571-0.722) and 0.639 (95% CI: 0.563-0.714), respectively.

### 3.4 Variable selection and nomogram development

**Figure 5:**
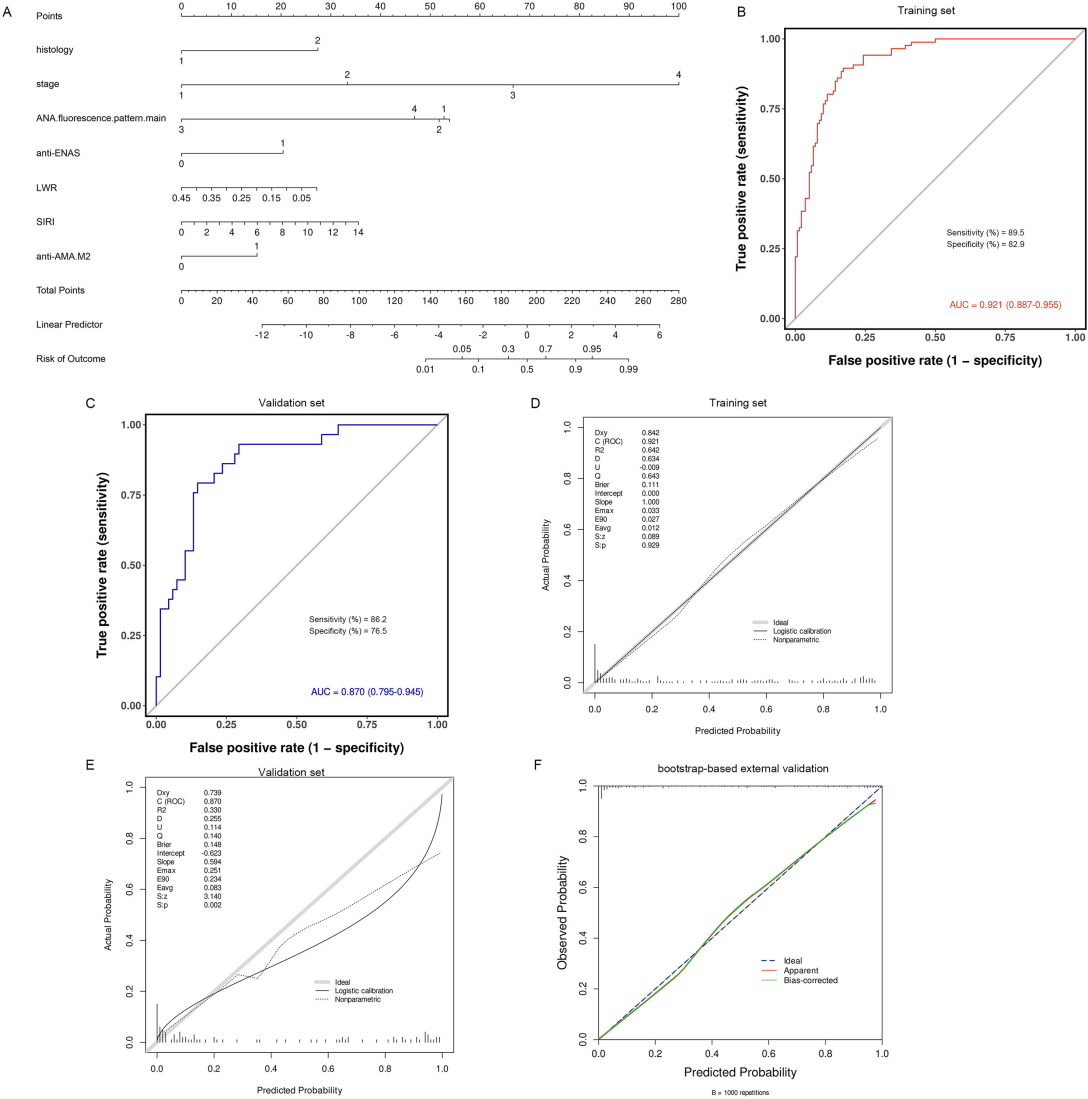
Nomogram for predicting bone metastasis in NSCLC and its performance evaluation. **(A)** Nomogram incorporating histology, stage, ANA fluorescence pattern, anti-ENAs, LWR, SIRI, and anti-AMA-M2. **(B)** Receiver operating characteristic (ROC) curve of the nomogram in the training set. **(C)** ROC curve of the nomogram in the validation set. **(D)** Calibration curve of the nomogram in the training set. **(E)** Calibration curve of the nomogram in the validation set. **(F)** Bootstrap-based calibration curve of the nomogram.

Based on LASSO regression, seven variables, namely histology, TNM stage, anti-ENAs, SIRI, LWR, anti-AMA-M2, and ANA fluorescence pattern, were selected and incorporated into a nomogram for individualized risk prediction (**Figure 5A**). No further variable elimination was performed based solely on multivariable *P* values, and all variables selected by LASSO were retained in the nomogram. The corresponding multivariable logistic regression coefficients and effect estimates for the variables included in the nomogram are presented in **Supplementary Table 3**. In the multivariable logistic regression analysis, anti-ENAs positivity was associated with increased odds of bone metastasis compared with anti-ENAs negativity (OR 5.16, 95% CI 2.09-12.74, *P*<0.001). TNM stage was also significantly associated with bone metastasis (OR 14.59, 95% CI 4.79-44.49, *P<* 0.001). Compared with squamous carcinoma, adenocarcinoma was significantly associated with a higher likelihood of bone metastasis (OR 4.64, 95% CI 1.99-11.31, *P*=0.005). By contrast, anti-AMA-M2 was not significantly associated with bone metastasis (OR 3.39, 95% CI 0.78-14.71, *P*=0.103). For ANA fluorescence pattern, using negativity as the reference category, nuclear granular pattern, cytoplasmic granular pattern, and other patterns were not significantly associated with bone metastasis, whereas nucleolar pattern was associated with lower odds of bone metastasis.

The predictive performance of the nomogram is shown in **Figures 5B, C** and **Supplementary Table 4**. ROC analysis demonstrated good discriminative ability for predicting bone metastasis, with an AUC of 0.921 (95% CI 0.887-0.955) in the training set and 0.870 (95% CI 0.795-0.945) in the validation set. The calibration plots of the nomogram for training set and the validation set are shown in **Figures 5D, E**, demonstrating good agreement between the observed and predicted bone metastasis. Both calibration curves closely follow the ideal line, indicating that the predicted probabilities are consistent with the actual outcomes in both cohorts.

Internal validation was conducted using bootstrapping with 1000 samples to assess the robustness of the prediction model. The corrected Harrell’s C-index of the nomogram model obtained from bootstrap resampling was 0.884 (**Supplementary Table 5**), indicating good internal validation. **Figure 5F** shows that the calibration curve constructed by bootstrap, apparent line and bias-corrected line deviated only slightly from the ideal line, indicating good concordance between the predictions and observations.

### 3.5 Predictive performance and clinical utility of the nomogram

**Figure 6:**
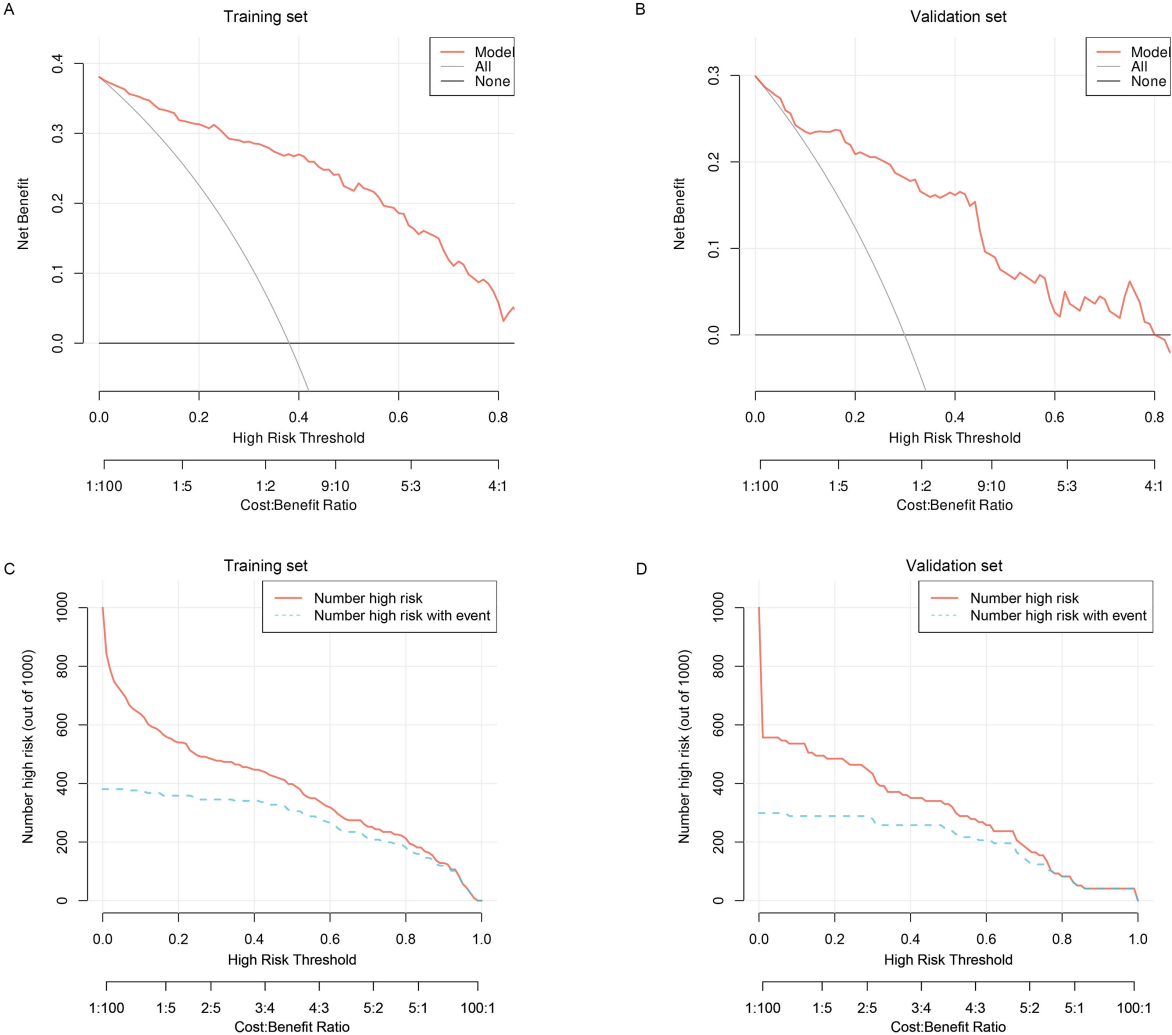
Decision curve analysis and clinical impact curves of the nomogram in the training and validation sets. **(A)** Decision curve analysis of the nomogram in the training set. **(B)** Decision curve analysis of the nomogram in the validation set. **(C)** Clinical impact curve of the nomogram in the training set. **(D)** Clinical impact curve of the nomogram in the validation set.

The DCA was performed to evaluate the clinical utility of the nomogram in both the training and validation sets (**Figures 6A, B**). The results showed that the nomogram provided a higher net benefit than the “treat-all” and “treat-none” strategies across a wide range of threshold probabilities, indicating favorable clinical usefulness for predicting bone metastasis in NSCLC. In both cohorts, the model maintained positive net benefit over most of the clinically relevant threshold range, with generally consistent performance in the validation set.

Clinical Impact Curves (CICs) were further generated to assess the practical impact of the nomogram (**Figures 6C, D**). In both the training and validation sets, the number of patients classified as high risk decreased progressively as the threshold probability increased. Meanwhile, the curve representing the number of high-risk patients with events remained relatively close to the total number classified as high risk at higher threshold probabilities, particularly when the threshold exceeded approximately 0.6, suggesting good concordance between model-predicted high-risk cases and actual bone metastasis events. These findings further support the clinical applicability and robustness of the nomogram.

### 3.6 Incremental predictive value of novel biomarkers over the baseline model

**Figure 7:**
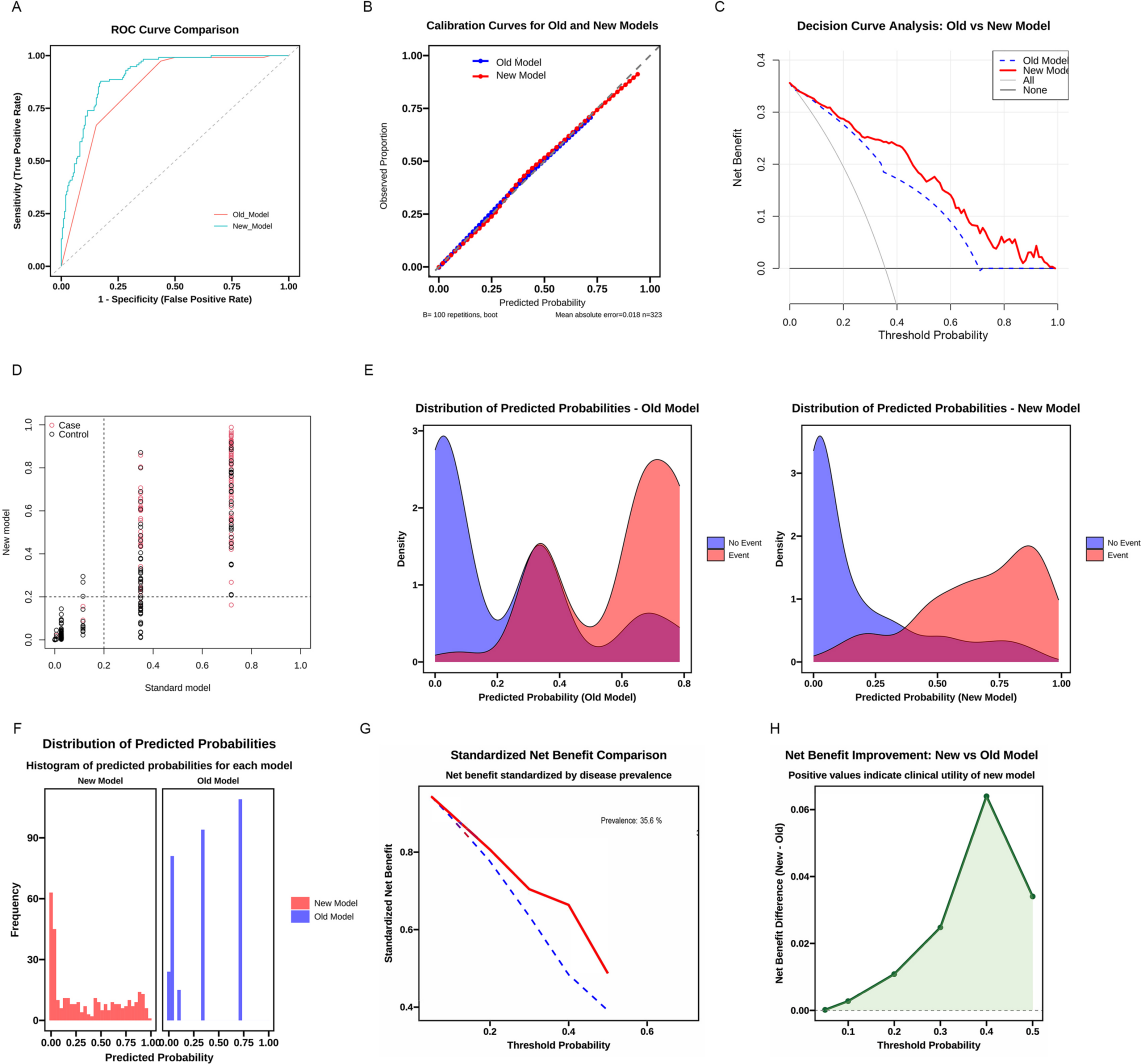
Comparison between the baseline model and the updated model. **(A)** Receiver operating characteristic (ROC) curves comparing the discrimination performance of the two models. **(B)** Calibration curves of the baseline (old) model and the updated (new) model. **(C)** Decision curve analysis comparing the clinical net benefit of the two models. **(D)** Reclassification scatter plot showing changes in predicted risk between the baseline and updated models. **(E)** Distribution of predicted probabilities for event and non-event groups in the baseline (old) model and the updated (new) model. **(F)** Histogram showing the distribution of predicted probabilities for the baseline (old) and updated (new) models. Red bars represent the updated model, and blue bars represent the baseline model. **(G)** Comparison of standardized net benefit between the two models across different threshold probabilities. **(H)** Net benefit difference between the updated model and the baseline model across threshold probabilities.

To clarify the incremental value of the newly added variables, two nested models were compared. The baseline model included only conventional clinicopathological variables, namely histology and stage, whereas the updated model additionally incorporated ANA fluorescence pattern, anti-ENAs, LWR, SIRI, and anti-AMA-M2. Compared with the baseline model, the updated model showed better overall predictive performance. It also had lower AIC, BIC, and deviance values, indicating improved overall model fit (**Supplementary Table 6**). The ROC curves showed that the updated model consistently outperformed the baseline model, indicating better discrimination (**Figure 7A**). Calibration plots demonstrated good agreement between predicted and observed risks for both models, with the updated model appearing slightly closer to the ideal reference line, suggesting modestly improved calibration (**Figure 7B**). Decision curve analysis further showed that the updated model provided a higher net benefit across a broad range of threshold probabilities, indicating superior clinical utility (**Figure 7C**).

To further assess the incremental predictive value of the newly added markers beyond the baseline model, continuous NRI and IDI were calculated. The continuous NRI was 0.822 (95% CI: 0.610-1.026, *P*<0.001), indicating significantly improved risk reclassification. The IDI was 0.121 (95% CI: 0.082-0.158, *P*<0.001), indicating a significant improvement in discrimination between subjects with and without the outcome (**Supplementary Table 7**). Together, these findings support the incremental predictive value of the newly added variables.

The reclassification scatter plot showed that the updated model reassigned predicted risks for a subset of individuals relative to the baseline model, indicating that the newly added predictors influenced individual risk estimation (**Figure 7D**). In the outcome-specific distributions of predicted probabilities, substantial overlap between event and non-event groups remained in the baseline model, whereas the updated model showed greater separation, with non-events concentrated more in the low-risk range and events more in the high-risk range (**Figure 7E**). In addition, the overall distribution of predicted probabilities was broader and more continuous in the updated model, suggesting finer risk stratification and more individualized risk estimation (**Figure 7F**).

At the clinical decision level, the standardized net benefit of the updated model was consistently higher than that of the baseline model across the evaluated threshold probabilities (**Figure 7G**, **Supplementary Table 8**). Moreover, net benefit difference analysis showed that the updated model provided a positive incremental net benefit throughout the assessed threshold range, with the greatest improvement observed at intermediate-to-high thresholds (**Figure 7H**, **Supplementary Table 8**).

Overall, these findings suggest that the addition of autoantibody and inflammation-related markers improves model performance beyond conventional clinicopathological factors and may enhance risk stratification for bone metastasis in patients with NSCLC.

## 4 Discussion

To provide preliminary biological context for the observed association between autoantibody-related markers and bone metastasis risk, we performed an exploratory analysis using GEO datasets comparing bone metastatic and non-metastatic NSCLC patients. Autoimmunity-related genes, including TRIM21, IRF7, CENPB, CD19, and other metastasis-associated genes, were significantly upregulated in the bone metastasis group. Functional enrichment analyses further indicated that these genes were primarily involved in immune activation and inflammatory signaling pathways, such as cytokine–cytokine receptor interaction, chemokine signaling, and JAK-STAT signaling, as well as processes related to leukocyte activation and inflammatory response.

In this study, we retrospectively analyzed the clinical and laboratory data of 323 patients with NSCLC to identify variables associated with bone metastasis and to develop a predictive nomogram. Based on LASSO regression in the training cohort, seven variables, namely histology, TNM stage, anti-ENAs, SIRI, LWR, anti-AMA-M2, and ANA fluorescence pattern, were selected and incorporated into the final nomogram. Multivariable logistic regression was subsequently used to estimate the effect sizes of these variables. These results provide a clinically actionable tool for individual risk stratification, which is crucial given the high prevalence and severe morbidity associated with metastasis in NSCLC (38, 39).

In this study, histology and TNM stage were among the variables selected by LASSO regression and incorporated into the final nomogram, and both remained significantly associated with bone metastasis in the multivariable logistic regression analysis. Compared with squamous cell carcinoma, adenocarcinoma was associated with a higher likelihood of bone metastasis, which is consistent with previous studies (40–42). As the predominant histological subtype of lung cancer, adenocarcinoma exhibits distinct metastatic biological behavior. Arising predominantly in the peripheral lung, these tumors are more prone to hematogenous dissemination and subsequent skeletal colonization, which may partly explain their stronger tendency toward bone metastasis compared with squamous cell carcinoma (43, 44). TNM stage was also significantly associated with bone metastasis in our study, with more advanced stage corresponding to a substantially higher risk. This finding is in agreement with previous reports (45). TNM stage reflects overall tumor burden and disease extent, both of which are closely related to metastatic potential. Patients with advanced-stage disease often show greater tumor heterogeneity, stronger invasive ability, more extensive vascular and tissue involvement, and enhanced secretion of chemokines and adhesion molecules, all of which may facilitate distant dissemination and bone colonization (46, 47). Therefore, histology and TNM stage remain important clinicopathological factors for risk stratification of bone metastasis in NSCLC.

The causes of autoantibody production in cancer patients are currently thought to include mutations in gene products, aberrant protein expression and post-transcriptional modifications, pro-immune environments, anticancer therapies, cross-reactivity of tumor-specific lymphocytes, epitope spreading, microbiota and genetic factors (48). Autoantibodies have been widely used as biomarkers for cancer risk assessment and adjunctive diagnosis in various malignancies (22, 23, 49, 50). However, it is not clear how the presence of autoantibodies in lung cancer patients affects the development of the tumor itself. In this study, ANA and anti-ENAs were analyzed in patients with NSCLC. In the multivariable logistic regression analysis, anti-ENAs positivity was associated with increased odds of bone metastasis, suggesting that autoimmune-related serological alterations may be involved in the metastatic process of NSCLC. In addition, ANA fluorescence patterns were also incorporated into the final nomogram, indicating a potential contribution of autoantibody-related features to risk stratification for bone metastasis. Nisihara et al. (51) reported that ANA positivity was significantly more frequent in patients with breast cancer than in those with benign lesions or healthy controls, with the nuclear granular pattern being the most common ANA immunofluorescence pattern, which is broadly consistent with our findings. Other studies have also suggested that tumor-associated autoantibodies may be related to malignant progression and prognosis (52, 53). These observations support the possibility that autoantibody profiles reflect alterations in the immune microenvironment associated with tumor progression. However, the biological role of autoantibodies in cancer remains complex and may vary across tumor types. To our knowledge, evidence regarding the relationship between anti-ENAs and bone metastasis in NSCLC remains limited. Therefore, our findings suggest that anti-ENAs and ANA-related features may provide additional value for risk stratification of bone metastasis in NSCLC, although the underlying mechanisms and external generalizability require further investigation.

Disturbed inflammatory responses play an important role in tumor initiation, progression, and metastasis (54, 55). In the present study, inflammation-related markers were incorporated into the final nomogram, including SIRI and LWR, suggesting that systemic inflammatory status may contribute to the risk stratification of bone metastasis in NSCLC. Although neither SIRI nor LWR reached statistical significance in the multivariable logistic regression analysis, both variables were retained by LASSO regression and included in the final model, indicating their potential incremental contribution to prediction when considered together with clinicopathological and autoantibody-related variables. SIRI is calculated as neutrophil count × monocyte count/lymphocyte count and reflects the balance between host inflammatory activation and antitumor immune response. Lymphocytes are essential for immune recognition, immune surveillance, and antitumor activity (58–60). By contrast, monocytes and tumor-associated macrophages derived from them can promote tumor angiogenesis, extracellular matrix remodeling, immune suppression, and metastatic spread (56–58). Neutrophils also participate in tumor-promoting inflammation and may facilitate invasion and dissemination through multiple mechanisms. Therefore, an elevated SIRI may reflect a protumor inflammatory microenvironment and impaired antitumor immunity. LWR, defined as the ratio of lymphocyte count to white blood cell count, may reflect the relative proportion of lymphocyte-mediated immune surveillance within the overall systemic inflammatory response (59). A lower LWR may indicate a weakened antitumor immune status and a relatively enhanced inflammatory burden, which could be associated with tumor progression and metastasis (60). Taken together, these findings support the notion that inflammation-based hematological markers may provide complementary information for assessing the risk of bone metastasis in NSCLC (61).

Based on these findings, we developed a nomogram incorporating histology, TNM stage, anti-ENAs, SIRI, LWR, anti-AMA-M2, and ANA fluorescence pattern for individualized risk prediction of bone metastasis in NSCLC. The model showed good discrimination, calibration, and clinical utility, and the addition of autoantibody- and inflammation-related markers improved performance beyond conventional clinicopathological factors alone.

Nevertheless, this study has several limitations. First, this was a single-center retrospective study with a relatively limited sample size, which may affect the stability and generalizability of the findings. Second, although multiple autoantibody-related variables were included, the scope of serological biomarkers remained limited, and other potentially relevant immune-related markers were not assessed. Third, this study focused mainly on routinely available hematological and serological indicators, while other potentially informative laboratory biomarkers were not comprehensively evaluated. Finally, the underlying biological mechanisms linking autoantibody profiles and inflammation-related markers to bone metastasis in NSCLC were not directly investigated. Therefore, larger multicenter studies and mechanistic investigations are warranted to validate and extend these findings.

## 5 Conclusion

In conclusion, a nomogram incorporating histology, TNM stage, anti-ENAs, SIRI, LWR, anti-AMA-M2, and ANA fluorescence pattern demonstrated good performance in predicting the risk of bone metastasis in patients with NSCLC. This model may serve as a useful tool for individualized risk assessment and risk stratification, with potential value in supporting clinical decision-making.

## Data Availability

The raw data supporting the conclusions of this article will be made available by the authors, without undue reservation.
