# Supplementary material for: Autoantibodies combined with systemic inflammation markers for predicting bone metastases in non-small cell lung cancer patients
Source: Front Immunol. 2026 May 26;17:1747604. doi: 10.3389/fimmu.2026.1747604 (PMC13246678; doi:10.3389/fimmu.2026.1747604)
Supplement: Supplementary file 1 [file Table1.docx]

**Supplementary Tables**

Table S1. Table of Assignments.

| Variables | Assign a value to something |
| --- | --- |
| Bone metastases | No = 0, Yes = 1 |
| Gender | Female = 0, Male = 1 |
| Smoking history | No = 0, Yes =1 |
| Histology | Squamous = 1, Adenocarcinoma = 2 |
| ANA | Negative = 0, Positive = 1 |
| ANA main fluorescent pattern titer | ﹤1:100 = 1, 1:100 =2, 1:320 = 3, 1:1000 = 4, 1:3200 = 5 |
|  | Negative = 0, Nuclear Granular Pattern = 1,  Cytoplasmic Granular Pattern = 2, Nucleolar Pattern = 3, Other s = 4 |
| anti-ENAs | Negative = 0, Positive = 1 |
| nRNP | Negative = 0, Positive = 1 |
| Sm | Negative = 0, Positive = 1 |
| SS-A | Negative = 0, Positive = 1 |
| Ro-52 | Negative = 0, Positive = 1 |
| SS-B | Negative = 0, Positive = 1 |
| Scl-70 | Negative = 0, Positive = 1 |
| Jo-1 | Negative = 0, Positive = 1 |
| CENP B | Negative = 0, Positive = 1 |
| PCNA | Negative = 0, Positive = 1 |
| ds-DNA | Negative = 0, Positive = 1 |
| nucleosomes | Negative = 0, Positive = 1 |
| histones | Negative = 0, Positive = 1 |
| RIB-P | Negative = 0, Positive = 1 |
| AMA-M2 | Negative = 0, Positive = 1 |

ANA: antinuclear antibody. anti-ENAs: antibodies against extractable nuclear antigens.

RIB-P: ribosomal P protein

Table S2. AUC Values and 95% Confidence Intervals for Variables.

| Variable | AUC Value | AUC 95% Confidence Interval |
| --- | --- | --- |
| histology | 0.733 | (0.673-0.793) |
| stage | 0.746 | (0.699-0.792) |
| ANA luorescence pattern main | 0.573 | (0.506-0.640) |
| LWR | 0.639 | (0.563-0.714) |
| anti-ENAS | 0.669 | (0.606-0.733) |
| anti-AMA-M2 | 0.560 | (0.517-0.603) |
| SIRI | 0.647 | (0.571-0.722) |

*Significant values (*P* < 0.05)

Abbreviations: CI = Confidence Interval, OR = Odds Ratio

SIRI: System inflammation response index. LWR: Leukocyte-to-White blood cell-related ratio. anti-ENAs: antibodies against extractable nuclear antigens.

Table S3. Multivariate Logistic regression for Internal Cohort.

| Characteristic | OR | 95% CI | *P* Value |
| --- | --- | --- | --- |
| anti-ENAS |  |  | <0.001 |
| 0 | — | — |  |
| 1 | 5.16 | 2.09, 12.74 |  |
| TNM stage | 14.59 | 4.79, 44.49 | <0.001 |
| histology |  |  | 0.005 |
| 1 | — | — |  |
| 2 | 4.64 | 1.99, 11.31 |  |
| anti-AMA-M2 |  |  | 0.103 |
| 0 | — | — |  |
| 1 | 3.39 | 0.78, 14.71 |  |
| ANA luorescence pattern main |  |  |  |
| 0 | — | — |  |
| 1 | 0.92 | 0.34, 2.45 | 0.860 |
| 2 | 0.85 | 0.17, 4.27 | 0.840 |
| 3 | 0.01 | 0.00, 0.16 | <0.001 |
| 4 | 0.57 | 0.10, 3.18 | 0.520 |
| LWR | 0.01 | 0.00, 10.62 | 0.187 |
| SIRI | 1.23 | 0.93, 1.62 | 0.153 |

Abbreviations: CI = Confidence Interval, OR = Odds Ratio

SIRI: System inflammation response index. LWR: Leukocyte-to-White blood cell-related ratio. anti-ENAs: antibodies against extractable nuclear antigens.

| Table S4. The prediction value of the nomogram model. | | |
| --- | --- | --- |
| Variables | Training set | Validation set |
| AUC | 0.921 | 0.870 |
| Sensitivity (%) | 89.5 | 86.2 |
| Specificity (%) | 82.9 | 76.5 |
| PPV (%) | 76.2 | 61.0 |
| NPV (%) | 92.8 | 92.9 |
| Accuracy (%) | 85.4 | 79.4 |

PPV: Positive predictive value. NPV: Negative predictive value.

Table S5. Discrimination performance of the generalized linear model validated by 1000-iteration bootstrap resampling (AUC, Sensitivity, and Specificity).

| parameter | ROC | Sens | Spec | ROCSD | SensSD | SpecSD |
| --- | --- | --- | --- | --- | --- | --- |
| none | 0.8844572 | 0.7557626 | 0.8398509 | 0.03064274 | 0.0894418 | 0.05709799 |

Table S6. Comparison of model fit statistics between the baseline and updated models.

| Model | AIC | BIC | Deviance |
| --- | --- | --- | --- |
| Old Model | 288.7247 | 300.0577 | 282.7247 |
| New Model | 258.2409 | 299.7951 | 236.2409 |

Table S7. Net reclassification improvement (NRI) and integrated discrimination improvement (IDI) for the updated model compared with the baseline model.

| Metric | Estimate | CI 95 lower | CI 95 upper | P value |
| --- | --- | --- | --- | --- |
| Continuous NRI | 0.8221572 | 0.61030545 | 1.0257156 | <0.001 |
| IDI | 0.1214876 | 0.08178827 | 0.1578309 | <0.001 |

Table S8. Comparison of net benefit between the baseline and updated models across different threshold probabilities.

| Threshold Probability | Old Model Net Benefit | New Model Net Benefit | Difference | Relative Improvement Percent |
| --- | --- | --- | --- | --- |
| 0.05 | 0.3359948 | 0.3361577 | 0.0001629461 | 0.0 |
| 0.10 | 0.3171655 | 0.3199174 | 0.0027519780 | 0.9 |
| 0.20 | 0.2763158 | 0.2871517 | 0.0108359133 | 3.9 |
| 0.30 | 0.2260062 | 0.2507740 | 0.0247678019 | 11.0 |
| 0.40 | 0.1723426 | 0.2363261 | 0.0639834881 | 37.1 |
| 0.50 | 0.1393189 | 0.1733746 | 0.0340557276 | 24.4 |
